# Cell surface sphingomyelin: key role in cancer initiation, progression, and immune evasion

**DOI:** 10.1186/s12944-021-01581-y

**Published:** 2021-10-31

**Authors:** Hatem Tallima, Hassan M. E. Azzazy, Rashika El Ridi

**Affiliations:** 1 Department of Chemistry, School of Science and Engineering, The American University in Cairo, New Cairo, Cairo, 11835 Egypt; 2 Zoology Department, Faculty of Science, Cairo University, Giza, 12613 Egypt

**Keywords:** Tumor, TAA, TSA, MHC class I, Natural killer cells, Sphingomyelin, Ceramide

## Abstract

Cell surface biochemical changes, notably excessive increase in outer leaflet sphingomyelin (SM) content, are important in cancer initiation, growth, and immune evasion. Innumerable reports describe methods to initiate, promote, or enhance immunotherapy of clinically detected cancer, notwithstanding the challenges, if not impossibility, of identification of tumor-specific, or associated antigens, the lack of tumor cell surface membrane expression of major histocompatibility complex (MHC) class I alpha and β2 microglobulin chains, and lack of expression or accessibility of Fas and other natural killer cell immune checkpoint molecules. Conversely, SM synthesis and hydrolysis are increasingly implicated in initiation of carcinogenesis and promotion of metastasis. Surface membrane SM readily forms inter- and intra- molecular hydrogen bond network, which excessive tightness would impair cell-cell contact inhibition, inter- and intra-cellular signals, metabolic pathways, and susceptibility to host immune cells and mediators. The present review aims at clarifying the tumor immune escape mechanisms, which face common immunotherapeutic approaches, and attracting attention to an entirely different, neglected, key aspect of tumorigenesis associated with biochemical changes in the cell surface that lead to failure of contact inhibition, an instrumental tumorigenesis mechanism. Additionally, the review aims to provide evidence for surface membrane SM levels and roles in cells resistance to death, failure to respond to growth suppressor signals, and immune escape, and to suggest possible novel approaches to cancer control and cure.

## Introduction

After decades of scientific investigations and billions of dollars, the option to the cancer drama remains surgery, provided the tumor is operable. The second option is radiation and chemotherapy, which potentially undermine the host immune responses. The third option is some attempts at personalized immunotherapy available uniquely at the most advanced centers in the developed countries and for the richest; however, with limited success rates [1]. Immunotherapy of cancer is predominantly a change of focus from direct targeting of cancer cells to generating tumor-reactive immune cells. Immune-therapy involves generation or activation of host immune effectors directed to tumor-specific (TSA) or associated (TAA) antigens, which are presented on the cell surface. Immunological approaches in cancer management that neglect lack of tumor cells surface membrane expression of TSA or TAA, MHC class I molecules, and natural killer (NK) cells activating checkpoints may not be effective [2].

**Fig. 1:**
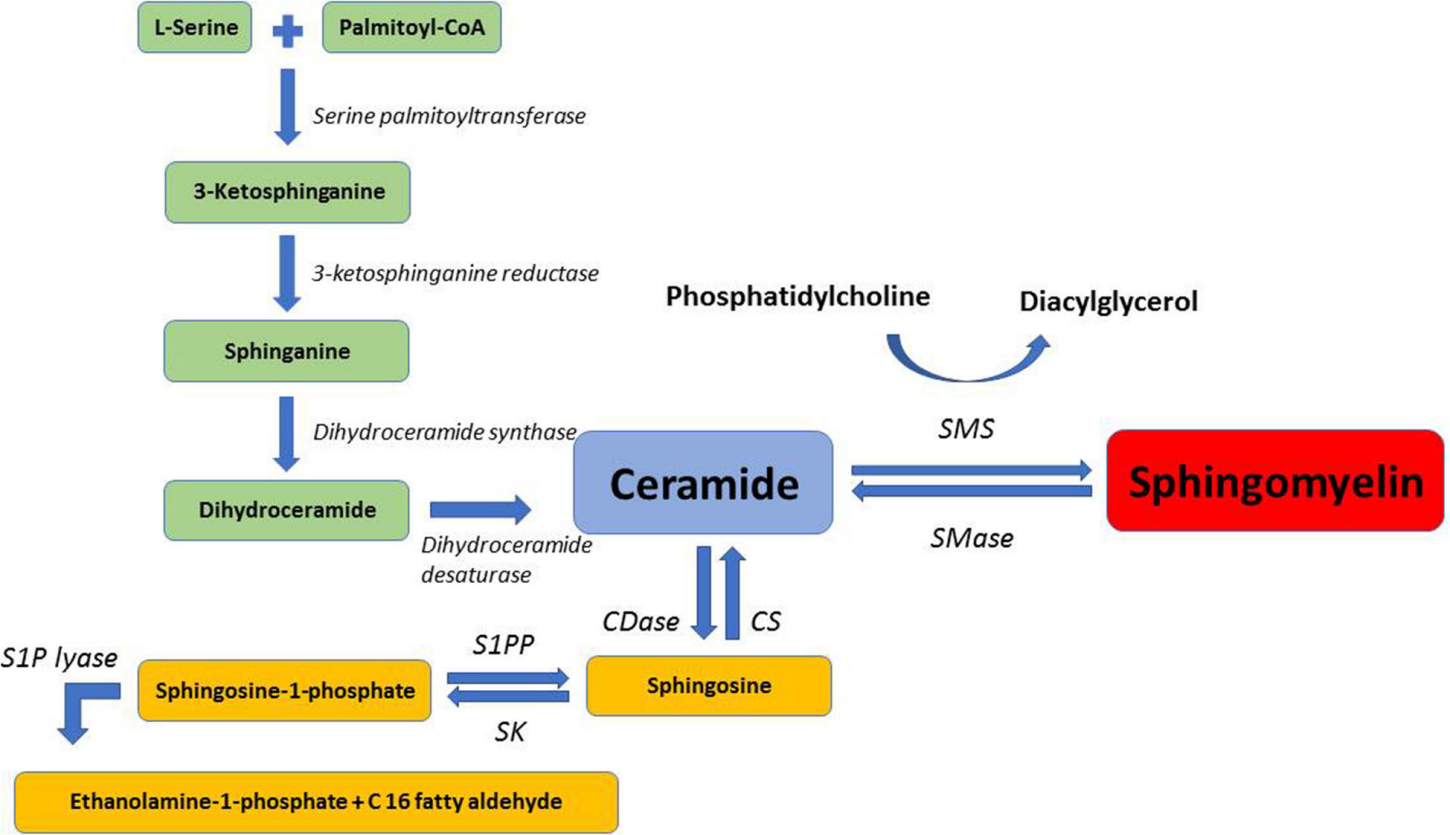
Major steps in sphingomyelin metabolism. SMS, sphingomyelin synthase; SMase, sphingomyelinase; CS, ceramide synthase; CDase, ceramidase; S1PP, sphingosine-1-phosphate phosphatidase; SK, sphingosine kinase; S1P, sphingosine 1-phosphate

Antigen presentation by tumor cells involves generation, proteasome proteolysis, entry into the endoplasmic reticulum for possible binding to HLA class I molecules, followed by transfer to the cell surface of a complex comprising a “putative” TAA or TSA [2]. The review challenges the existence of such antigens and accessibility of tumor cells surface MHC class I and NK cell activating molecules, thus precluding antigen presentation and preventing any immune attack mode on the cancer cells. Conversely, the review highlights the most fundamental concept of “contact inhibition”, now largely ignored, and which refers to contact-mediated inhibition of locomotion, migration, and proliferation when normal cells come in contact with one another [3]. Failure of contact inhibition is one of the major mechanisms underlying the initiation of tumorigenesis and is certainly the responsibility of the cell surface phospholipids, cholesterol, and sphingomyelin (SM). Therefore, attention is herein directed to the cell surface biochemical and biophysical changes in SM levels and instrumental roles in cancer initiation, growth, and metastasis (Fig. 1). The release of diacyl glycerol upon SM synthesis is clarified in Fig. 1. This molecule is central to a too large plethora of metabolic and signaling pathways, and its role in tumorigenesis encompasses several axes, and is not restricted to the content of plasma membrane SM.

## Tumor immune evasion

### Tumor-associated and tumor-specific antigens

Many tumor-associated (TAA) and tumor-specific (TSA) antigens are proteins expressed in fetal and normal adult tissues and stressed cells, found to be upregulated in cancer cells and serum of cancer patients (Table 1). All these molecules are actually self-antigens which may not induce immune responses specific to the tumor cells, even if they are displayed on the apical surface. They have diagnostic and prognostic value, but may not be instrumental in induction of immune effectors against cancer cells [4–12]. The extreme difficulty and challenges in identifying genuine TAA or TSA, which possess the needed specificity and immunogenicity, were recently emphasized [13–16].

**Table 1:** Tumor-associated and tumor-specific antigens

Marker	Full name	Tissue	Malignancy	Ref
AFP	Alfa fetoprotein	Fetal liver	Liver, gut, ovaries	[4]
CEA	Carcinoembryonic antigen	Blood	Gastric, lung	[4]
HSP	Heat shock proteins	Stressed cells	lung, gut, prostate	[4]
CA	Carbohydrate antigens	All cells	Gastric, lung, pancreas	[4]
MUC1	Mucin 1	Epithelial cells	Lung, breast, pancreas	[4]
PSA	Prostate-specific antigen	Prostate	Prostate	[4]
MAGE	Melanoma-associated antigen	Testis	Lung	[4, 5]
NY-ESO-1	Cancer/testis antigen	Testis	Esophagus	[6–8]
SSX-2	Cancer/testis antigen	Testis	Various cancer	[7, 9, 10]
GPC3	Glypican-3	Fetal and adult	Liver, lung, melanoma	[4]
Midkine	Growth-promoting factor 2	All cells	Nervous system	[7, 11]
EpCAM	Cell adhesion molecule	Epithelial cells	Epithelial carcinomas	[7]
PRAME	Preferentially in melanoma	Testis	Lung	[5, 7, 8]
WT1	Wilm’s tumor protein1	Urinogenital	Kidney	[7, 8]
Survivin	Inhibitor of apoptosis	All cells	Bladder	[7, 8, 12]

### Immunogenicity of the elusive tumor-associated antigens

**Fig. 2:**
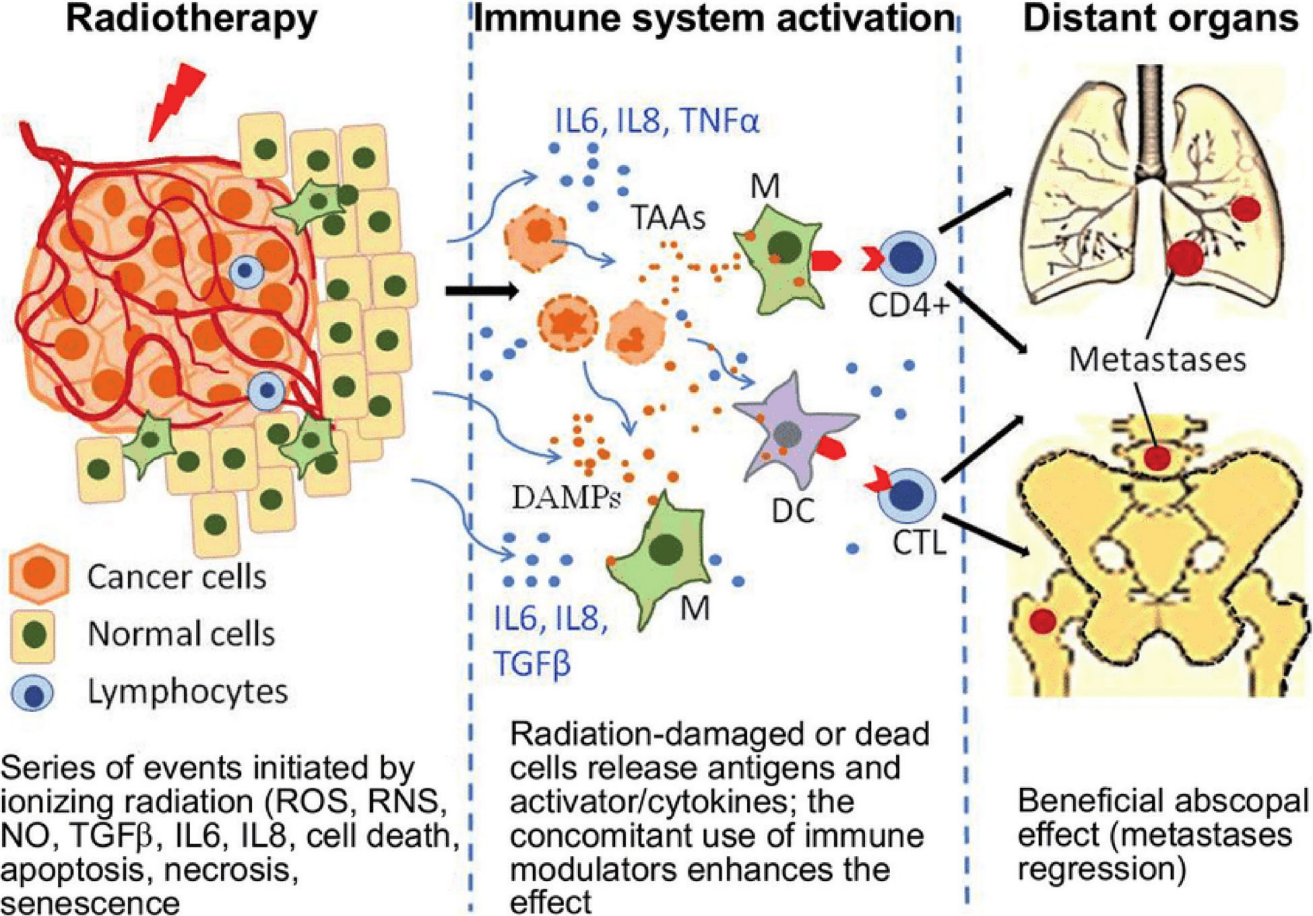
The abscopal effect. Irradiation of tumor cells results in the release of a plethora of danger associated molecular patterns (DAMP), which interact with innate immunity receptors on nearby cells, thus inducing the release of tumor necrosis factor alpha (TNFα), interleukin (IL) 6, transforming growth factor beta (TGFβ), the chemokine IL8, reactive oxygen (ROS) and nitrogen (RNS) species, and nitric oxide (NO). These inflammatory molecules provide the necessary signals for the recruitment and stimulation of dendritic cells (DC) and macrophages (M) to properly present TAA to helper (CD4+) and cytotoxic T (CTL) lymphocytes, which may target and kill residual and metastatic tumor cells bearing TAA on their surface membrane. The figure is uploaded on the net by Maria Widel and was reproduced with her permission [18]

A putative TAA released by proliferating or dying tumor cells would be presented by macrophages or dendritic cells to stimulate CD4+ and then CD8+ T lymphocytes and B cells, provided proper co-stimulatory signals are available. That is not the case in numerous situations, as protein antigens fail to stimulate innate immunity receptors with consequent inflammation and pain, which are often not perceived during tumor initiation and growth [2]. The importance of these signals is shown by the phenomenon termed abscopal effect, whereby some immunogenic tumors regress following distant thermal or irradiation intervention, which results in release of endogenous damage-associated molecular patterns [17–19]. The abscopal effect would certainly be effective provided tumors are able to display surface membrane-located TSA or TAA (Fig. 2).

### Humoral antibody targeting of putative TAA-bearing tumor cells

**Fig. 3:**
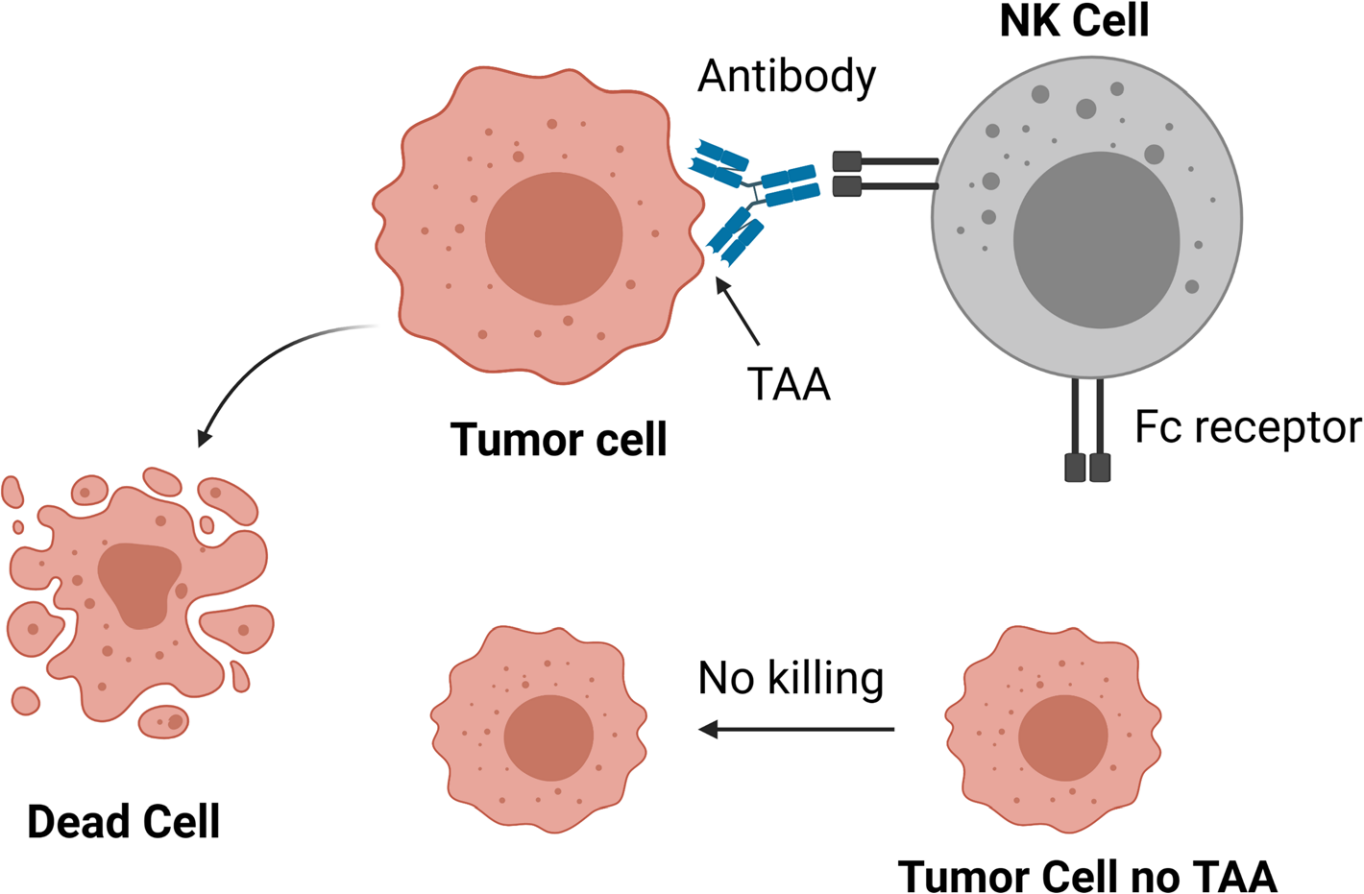
Antibody-dependent cell-mediated cytotoxicity (ADCC). Natural killer cells (NK) killing of tumor cells relies on their expression of surface membrane tumor-associated antigen (TAA). Drawn using BioRender

A TAA would then be the target of specific antibodies. If IgM or IgG1 specific antibodies are generated and access that tumor specific surface antigen, the complement system could be activated with limited effect on the tumor cell per se, but with considerable impact on the tumor microenvironment and on the host, if such antigen is shared with normal cells, especially in the lung and kidney. Antibodies that mediate natural killer (NK) cells and macrophage killing are more effective in tumor eradication, provided that antigen is expressed on the cell surface membrane (Fig. 3), and is specific to the tumor cells, not present as well on the surface of healthy epithelial, nervous system, and urogenital cells [4–12]. In fact, immunotherapy-generated antibodies will intensely engage with live or dying tumor cells-excreted and secreted products leading to their opsonization, while sparing the tumor cells.

### Cytotoxic CD8+ cells targeting of tumor cells and the lack of surface membrane MHC class I molecule expression

**Fig. 4:**
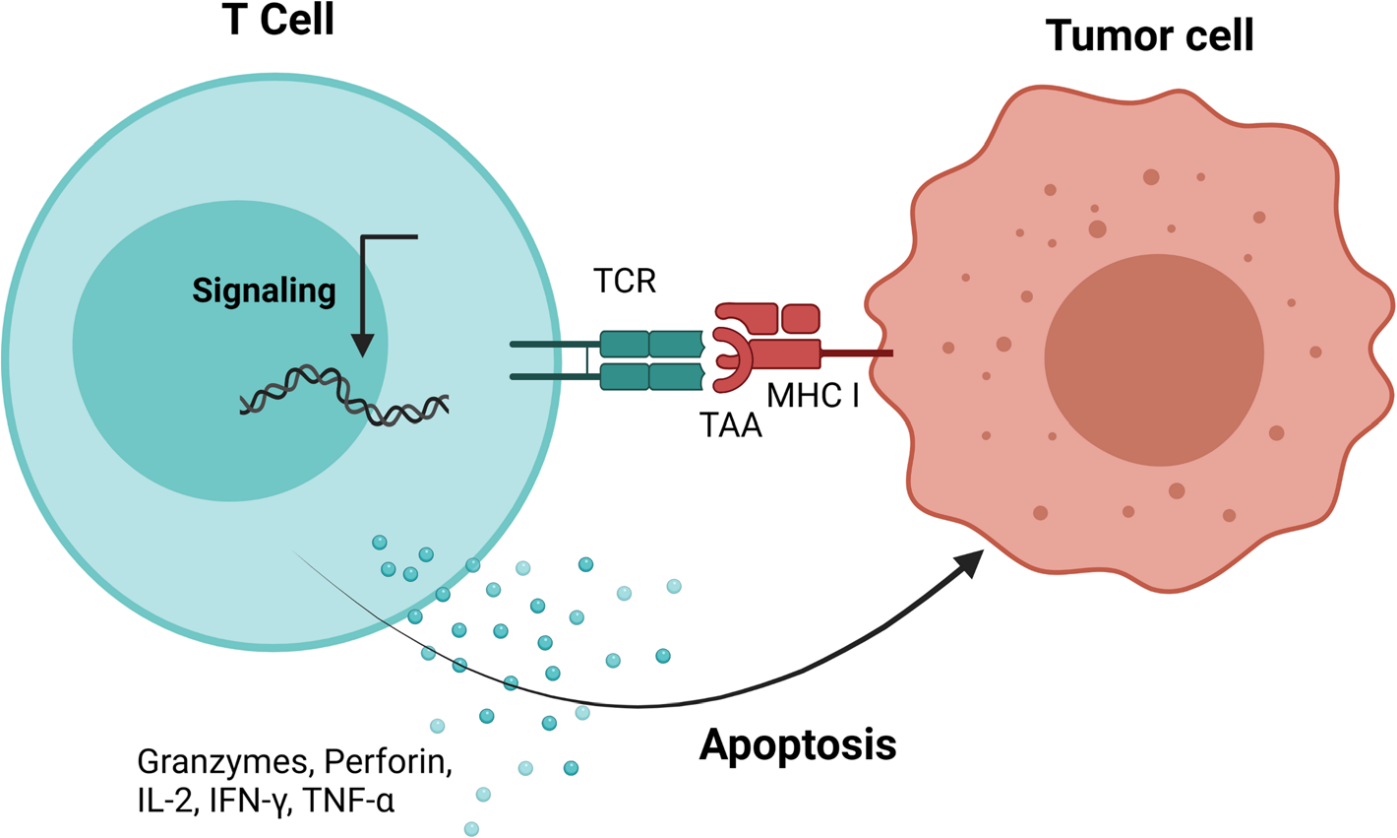
Cytotoxic T lymphocyte killing of tumor cells. Presentation of peptide TAA-derived peptides by surface membrane MHC class I molecule is mandatory for mediating killing by cytotoxic T cells. TCR, T cell receptor, IFN-γ, interferon gamma. Drawn using BioRender

Specific CD8+ cytotoxic lymphocytes would be life-saving if such putative TAA-derived peptides are presented on the surface of the tumor cells in association with MHC class I molecules (Fig. 4). Loss of surface membrane MHC class I molecule expression on cell surface membrane has, however, been documented for almost all tumors, and was found to be associated with a more malignant phenotype [**for review** [20–23]].

### Interaction of natural killer cells with tumor cells

**Fig. 5:**
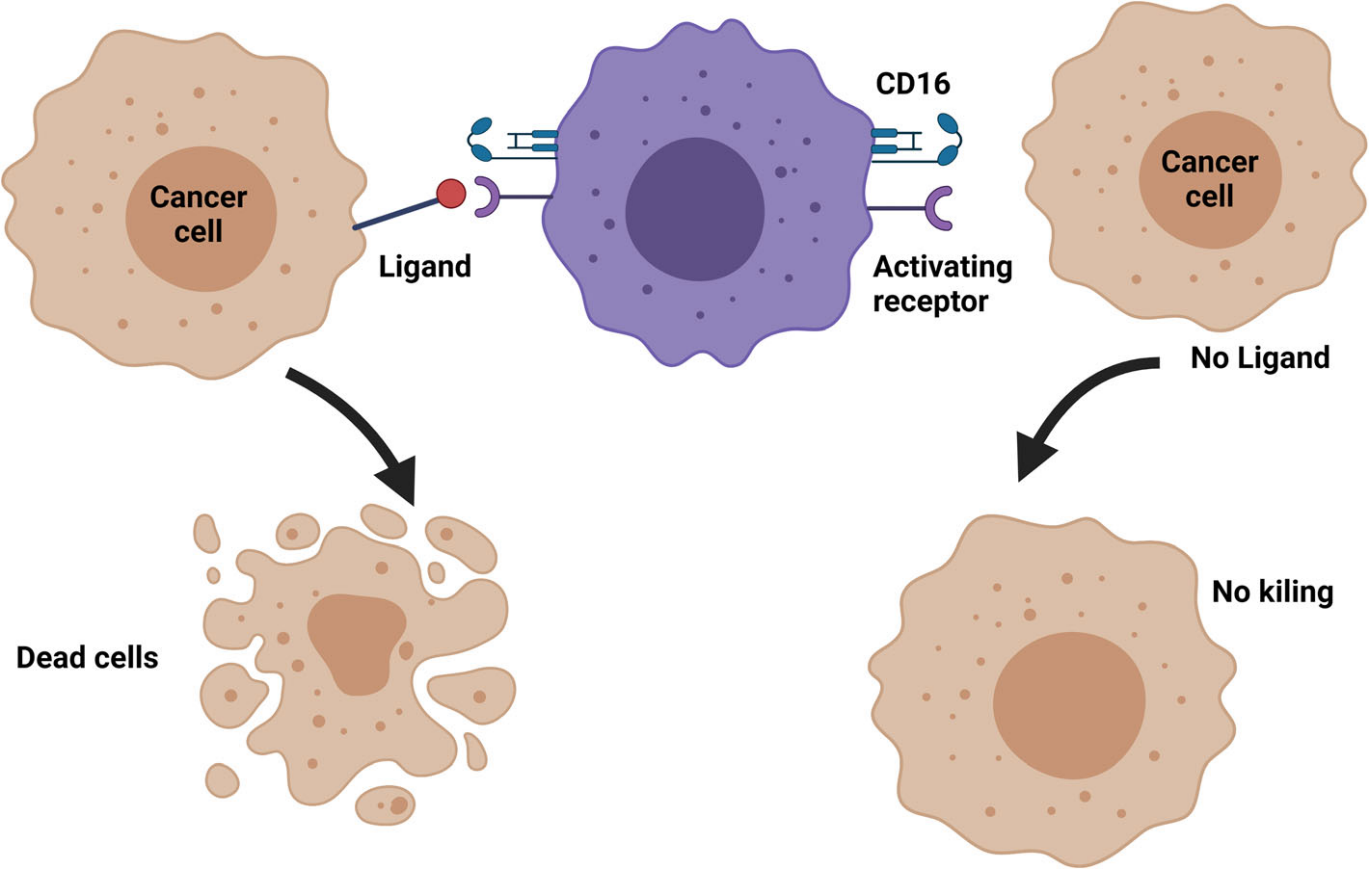
Natural killer cell killing of tumor cells. Natural killer (NK) cell kills MHC class I negative tumor cells if they express surface membrane NK cell-activating ligand. Drawn using BioRender

Despite such bleak scenario, it is fortunate that tumor cells displaying decrease or absence of surface membrane MHC class I molecules, invite NK cells into action, provided tumor necrosis factor (TNF) apoptosis-inducing receptor, Fas (also termed CD95 or Apo-1 or TNFRSF6) [24] and death-inducing receptors, TRAIL (TNF-related apoptosis-inducing ligand)- R1 (death receptor (DR)4 and TRAIL-R2 (DR5) [25, 26], and NK cell-activating molecules [27, 28] are displayed on the surface of the cancer cell (Fig. 5). It is likely NK cell-mediated killing mechanisms are instrumental in immune surveillance responsible for elimination of tumors during human life span [27–29]. If age, hormonal, neural, biochemical and immune factors lead to NK cell activity impairment, cancer cells would overgrow, and gradually show loss of accessibility of Fas and NK cell activating molecules. Several cancer cell lines showed little surface membrane Fas expression even after treatment with interferon-gamma (IFN-γ) [30]. Additionally, several reports have documented cancer cell surface membrane aberrant expression of ligands to NK cell activating and inhibitory receptors [31–33].

### The cell surface changes as initiators of carcinogenesis

In case of tumor cell surface membrane aberrant expression of TSA or TAA, MHC class I, and NK cells activating molecules, immunotherapeutic approaches that manipulate number and activity of antibody, CD4+, CD8+, NK cells and macrophages are of limited effect. The target of intervention should be the reason(s) behind decrease or lack of antigen expression, and most importantly, accessibility on the surface of cancer cells. Altered expression of surface membrane molecules has been ascribed to loss or reduction of gene expression, post translational modifications that prevented anchoring in surface membranes, hiding due to changes in the biochemical composition of cancer cells, or defects in the plasma membranes of cells that likely lead to loss or decrease of antigen exposure [20–23]. It is the biophysics and biochemistry of the cell surface that must be thoroughly examined in view of finding reliable solutions [34–39]. Changes in cell surface composition, electrical charge, and activity may be the reason for the loss of contact inhibition, which is directly related to the uncontrolled tumor cell proliferation [34–39]. The high net negative charge on the surface of tumor cells was ascribed to increase in phospholipids and was found to be associated with higher metastatic potential [38, 39].

### Increase in surface membrane sphingomyelin content leads to immune escape, and tumorigenesis initiation and progression

Remarkably, SM is apt at interacting with neighboring molecules and surrounding water molecules to generate a network of hydrogen bonds, via forming intermolecular hydrogen bonds between the glycosyl head groups, the amide and hydroxyls of the sphingosine base and of the hydroxy fatty acid. This extensive intermolecular hydrogen bonding capacity is a characteristic feature that distinguishes sphingolipids from the major lipid family in animal cells, the glycerolipids. These cannot form interlipid hydrogen bonds between their diglyceride moieties. The ester and ether groups can function only as hydrogen bond acceptors, not as donors [40–44]. Abnormal increase in SM content in surface membrane apical layer reduces membrane fluidity and permeability, and increases its rigidity and strength, leading to loss of contact inhibition and self-control mechanisms, decrease in cell to cell communication, reduced or inhibited cell surface molecules expression and signaling pathway coordination, and uncontrolled proliferation [34–39].

Following initiation of tumorigenesis, continuous increase in SM content and distribution on the cell surface membrane leads to progressive immune evasion from host effector cells and molecules, allowing tumor growth. The critical importance of SM metabolism in cancer progression is additionally due to its role in ceramide (Cer) production, because reduced SM degradation leads to decreased production of ceramide, an important signaling molecule for cancer biology including apoptosis, cell proliferation, cell migration, senescence, and inflammation [45–47]. Indeed, resistance to apoptosis is reported as one important mechanism by which tumor cells escape the action of potential immune effectors [45–51].

### Evidence for the role of surface membrane sphingomyelin in tumorigenesis

Several lines of research documented the role of surface membrane SM in tumorigenesis. First, changes in sphingolipids levels were established for several tumor cells and cell lines [52–57]. More specifically, level of SM in the outer leaflet of cell plasma membrane was reported to be significantly elevated in highly metastatic human prostatic adenocarcinoma cell lines compared to the lower metastatic variant [52]. Second, SV40-transformed human lung fibroblasts synthesized SM at an abnormally fast and high rate compared to untransformed cells [58]. Third, accumulating evidence suggest that colon, prostate, and kidney cancer are also associated with alterations in sphingolipids and their metabolizing enzymes [48, 59]. Fourth, high SM synthase expression and aberrant SM contents were associated with breast cancer progression and metastasis [60]. Fifth, increased SM content in frozen tissue samples of primary lung adenocarcinoma obtained from patients who underwent radical surgery was the most reliable indicator of recurrence, i.e., of degree of malignancy [61]. Six, low levels of surface membrane-associated neutral sphingomyelinase (nSMase)-2, responsible for SM hydrolysis, were associated with early recurrence of hepatic cell carcinoma after surgery [62]. Additionally, human hepatic cancer cell lines, HepG2 and Huh-7, were incubated with lysenin in indirect membrane immunofluorescence (IF) assays. Lysenin is SM-specific binding protein from the earthworm *Eisenia foetida*, which acts as a specific cytochemical probe for SM [63]. Intense surface membrane IF was displayed by HepG2 >> > Huh-7 Cell lines > non-cancerous cells. These results confirmed the presence of inordinate SM amount on the surface membrane of the liver cancer cells and its association with level of malignancy [64]. Indeed, sphingolipids control of the balance in cells between proliferation and apoptotic cell death has been amply documented [58].

### Aspects of altered sphingomyelin metabolism in cancer

Sphingomyelin is the most abundant sphingolipid in animal cell membranes, localized to the outer membrane leaflet where it greatly contributes to the formation of specialized liquid-ordered domains called lipid rafts. Sphingomyelin synthase (SMS) isoforms activity contribute to increased SM content and trafficking to the cell membrane. The activity of sphingomyelinase enzymes, notably neutral sphingomyelinase (nSMase)-2, generally localized at the plasma membrane, modulates the content of surface membrane SM via mediating its hydrolysis to ceramide and phosphocholine [40–47]. Altered sphingolipid metabolism was predicted to occur in early stages of oncogenic transformation, independently of genetic mutations, because sphingolipid metabolizing enzymes are rarely mutated [56].

Sphingomyelin metabolism in cancer cells was characterized by changes in SMS expression and activity [58]. Increased SMS expression in breast [60] and ovarian [65] cancer could be conducive to tumor metastasis. Depletion of SMS isoform 2 suppressed survival, growth, and migration of ovarian cancer cell lines, via disruption of lipid metabolism and mitochondrial function, and increase in oxidative damage [66].

Losses, mutation, and poor expression of the gene encoding nSMase were prevalent in breast and prostate cancer, and osteosarcoma cell lines [67, 68]. Loss, reduced or aberrant expression of nSMase has been reported in colorectal, gastric, and lung cancers, lymphomas, and acute myeloid and lymphocytic leukemia [69]. Notably, gene encoding nSMase-2 was hypermethylated and silenced in hepatic cell carcinoma, whereby gene overexpression elicited diminished cellular proliferation by 50%, and knockdown promoted tumor invasiveness and migratory capacities [62]. Hypermethylation or low expression of nSMase2-encoding gene were common events in oral squamous and renal cell carcinoma, associated with spread of tumor cells, larger tumor, higher malignancy grade, and earlier recurrence [70, 71].

Reduction in the potency of nSMase-mediated SM degradation pathway would lead to its accumulation at the apical lipid leaflet, excessive tightening of the SM-based hydrogen bond barrier and, consequently, impairment of cell-cell and cell-matrix interactions [40–44]. Marchesini et al. [72] discovered that overexpression of nSMase 2 expression causes confluence-induced growth arrest in breast cancer cells. Changes in cell nSMase2 expression, level, or activity likely led to primary tumor growth via SM accumulation on the cell membrane outer leaflet, thus interrupting cell-contact inhibition, and preventing exposure of cell surface membrane immune check points [64]. Recently, surface membrane excessive SM content in HepG2 and Huh-7 hepatic tumor cells was shown to be associated with decrease in nSMase activity. Triton-soluble surface membrane molecules of tumor and non-cancerous cells were assessed for nSMase activity using the Sphingomyelinase Amplex Red Assay of Invitrogen. The results of three independent assays revealed that nSMase activity of Hep G2 and Huh-7 tumor cells was 32 and 28% lower (*P* < 0.05) than that of normal cells, respectively. The study implicated reduced nSMase activity as responsible for high SM content in tumor cells surface membrane [64].

Ceramide is the central molecule in SM synthetic and hydrolysis pathways, and plays an important role in cancer metabolism. Balance between levels of the anti-proliferative Cer and the pro-survival action of its metabolite, sphingosine-1-phosphate, determines cell fate, and hence termed the sphingolipid rheostat of cancer cells [73]. A major mechanism of Cer generation involves hydrolysis of SM by neutral, acid, and alkaline sphingomyelinases, the former being associated with the cell plasma membrane and responsible for the control of surface membrane SM content [45–47, 49–51]. The levels of SM and Cer were up- and down-regulated, respectively in hepato cellular carcinoma tissues [74]. The six ceramide synthases genes were differently expressed in colorectal carcinoma, and overexpression led to impairment of the in vitro viability of cancer cell lines [75]. In support, alkaline ceramidase was found to be overexpressed in hepato cellular carcinoma tissue and cell lines, promoting cell proliferation via mediating Cer hydrolysis [76]. Breast cancer aggressiveness and proliferation were attributed to suppression of apoptosis via a Cer-associated pathway [49]. Synthesis and accumulation of Cer mediate cancer cell death via apoptosis, necroptosis, and autophagy [45, 47, 77].

### Role of surface membrane sphingomyelin in metastasis and drug resistance

Sphingomyelin is particularly enriched in tumor-derived exosomes, which contribute to cancer angiogenesis, invasion, metastasis, and drug resistance via shuttling anti-cancer drugs out of the tumor cells [78–81]. High SM content in cancer cell surface membrane reduces anti-cancer drug influx, interferes with endocytosis of nanoparticles-based drug delivery systems, and mediates drug sequestering in intracellular vesicles, major mechanisms in cancer drug resistance [82]. Content of surface membrane SM was shown to dictate the uptake level of the anti-pancreatic cancer drug, gemcitabine [83]. Cancer cells cloaked in SM-rich plasma membrane, surrounded by a tight hydrogen barrier made by interaction of SM with water molecules may traffic unscathed in blood vessels and capillaries, because host immune cells and effectors are prevented from accessing surface membrane immune checkpoint molecules, explaining the readiness of cancer cells to metastasize to distant locations [84].

### Impact of cancer therapy on surface membrane sphingomyelin content

Exposure to ionizing irradiation led to rapid nSMase-mediated hydrolysis of SM to Cer, and cancer cell death [85]. Resistance to traditional cancer treatments like cisplatin or irradiation was associated with nSMase inhibition, leading to SM accumulation and low Cer levels [86]. Conversely, etoposide used for the treatments of lung, testicular and ovarian cancer, lymphoma, leukemia, and neuroblastoma, and cytarabine used to treat acute myeloid leukemia, acute lymphocytic leukemia, chronic myelogenous leukemia, and non-Hodgkin’s lymphoma promote nSMase activation, leading to reduced surface membrane SM content and Cer accumulation [46, 47, 69]. The phospholipid analogue miltefosine, which has been approved for the treatment of breast cancer metastasis, and is currently used for the treatment of cutaneous metastases of mammary carcinoma was shown to significantly inhibit SM biosynthesis in human hepatoma and other tumor cells [87], promote efflux of cholesterol and SM from the surface membrane [88], and modulate membrane physical properties [89]. The anti-cancer drug daunorubicin induced specific activation of nSMase-2 in the human breast cancer cell line MCF-7, leading to depletion of surface membrane SM and accumulation of intracellular Cer [90]. Arsenic trioxide anti-cancer activity against human multiple myeloma and gastric cancer cell lines appeared to be attributed to alterations in the sphingolipid pathway [91]. Gentamicin induction of delayed cell growth and cell death of Hodgkin’s T-cell human lymphoblastic lymphoma acted via SM metabolism, notably nSMase stimulation in whole cells, increase in SM levels in nuclear but not outer membrane [92], and increase in cell surface elasticity [83]. Inhibition of cell proliferation and induction of apoptosis in myeloid, lymphoid, and solid cancer cell lines by Withanolide D isolated from the herb *Withania somnifera*, root extracts from *Panax ginseg*, or propolis-derived caffeic acid phenethyl ester were attributed to nSMase activation**-**mediated production of apoptotic Cer from membrane SM [93–95].

## Manipulating sphingomyelin metabolism for cancer control

Targeting SM metabolism may represent viable target for cancer cure [96–98]. This is supported by multiple reports. Blocking SM synthesis was recently found to increase immune responses to hepatic cell carcinoma, mantle cell lymphoma, and glioblastoma [99–101]. Additionally, therapeutic treatments that increased SM hydrolysis via activating the surface membrane-associated nSMase allowed tumor cell apical membrane antigens exposure to the host immune effectors and prevention of an instrumental immune escape mechanism, together with accumulation of intracellular Cer [46, 69]. Interference with SM metabolism was exploited to overcome multidrug resistance [49, 50, 102]. Specifically, inhibitors of sphingosine kinase were proposed for cancer treatment via increasing Cer levels [73].

### Surface membrane sphingomyelin-based prophylactic and therapeutic approaches to cancer

**Fig. 6:**
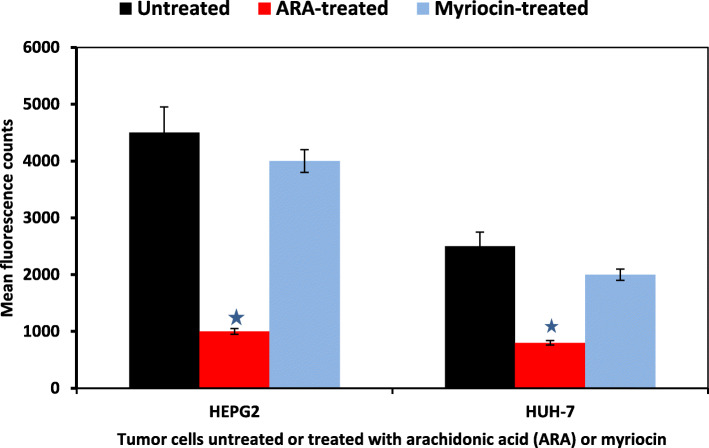
Sphingomyelin metabolism and proliferative capacity of hepatic tumor cells. Mean fluorescence counts in hepatic tumor cells quantitatively assayed for proliferation by the Alamar blue microplate test, and showing significant (*P* < 0.005, ★) decrease following exposure to arachidonic acid (ARA), a known activator of nSMase, which leads to SM hydrolysis and release of intracellular apoptotic ceramide, reflected in cell viability and proliferation decrease. Myriocin interferes with SM synthesis via blocking the initial step in the de novo synthesis of ceramide, leading to reduction of SM content in the cell membrane, and reduced proliferation.

The alkyl-lysophospholipid analogue edelfosine, resveratrol, miltefosine, and inhibitors of SM metabolism enzymes were used to alter tumor cell membrane fluidity and permeability and showed remarkable efficacy in preventing tumor progression [54, 85, 103, 104]. Devising novel anticancer strategies based on the modulation of lipid metabolism and the composition of the cell membrane was recently advised for the treatment of cancer and overcoming drug resistance, pioneering a novel field named membrane-lipid therapy [104–106]. Combination of chemotherapeutic drugs and Cer is presently increasingly used for tumor treatment [47, 51, 107–109]. Increasing the activity of nSMase 2 led to overcoming immune escape in melanoma cells [110]. Arachidonic acid (ARA), a potent nSMase activator, was shown to attenuate leukemia-derived HL-60, gastric, prostate and breast cancer cells growth via nSMase activation pathways [111–113]. Arachidonic acid was advocated as having the potential to increase the efficacy of currently used glioma treatments via its activation of nSMase [114]. Additionally, activation of HepG2 and Huh-7 tumor cells with ARA led to considerable decrease in surface membrane SM content as assessed using the lysenin test, increase in nSMase activity as judged by the Sphingomyelinase Amplex Red Assay, likely mediating highly significant (*P* < 0.0001) reduction in HepG2 and Huh-7 tumor cells proliferation level in the Alamar blue test (Fig. 6) [64]. These results are in entire accord with the reports documenting ARA selective tumoricidal action [115, 116].

## Study strength and limitations

In this review the role of the cell surface membrane SM in tumor initiation, progression, and metastasis was emphasized for the first time. Proposals in this review are directly and indirectly supported by a large number of published studies and findings. The review is, however, limited by the lack of experiments and trials dedicated to the assessment of the validity and efficacy of anti-tumor therapies based on reducing the level and distribution of sphingomyelin in experimental animals and humans.

## Conclusions and further perspectives

Cancer that becomes clinically detectable has evaded immunological surveillance, possibly due to failure of properly exposing cell surface membrane molecules, which are instrumental in interaction with the host immune effectors. Immunotherapy strategies may, therefore, not be of significant use. Future cancer management strategies should be directed at elucidating the fundamental flaw leading to low or absent surface membrane expression of molecules critical for tumor eradication. The review proposes that cell surface membrane SM accumulation, propensity to form intermolecular hydrogen bond barrier with adjacent molecules and surrounding water, and impairment of a major generation mechanism of the pro-apoptic Cer are instrumental in tumor initiation, progression, and metastasis. Innumerable published findings, reports, and studies supporting the hypothesis were presented. The recent progress in membrane-lipid therapy was delineated. Treatment with ARA, the membrane-associated nSMase-2 powerful activator, was proposed for prevention of tumor cells immune evasion and cancer management via hydrolysis of surface membrane SM, allowing proper cell-cell-contact inhibition, exposure of immune check molecules, and release of vigorous apoptotic signals. Experiments will be designed to assess the impact of ARA and other molecules capable of modulating the cell surface SM content in preventing tumor initiation, reversing tumor growth, and preventing cancer cells locomotion, migration, and metastasis in experimental hosts. Possible safe and highly efficacious prophylactic and therapeutic agents and molecules will be proposed for pre-clinical trials in experimental hosts and humans.

## Data Availability

Not Applicable. Data sharing is not applicable to this article as no datasets were generated or analyzed during the current study.

## Abbreviations

SM: Sphingomyelin
MHC: Major histocompatibility complex
TAA: Tumor-associated antigen.
TSA: Tumor-specific antigen
NK: Natural killer
TNF: Tumor necrosis factor
IFN-γ: Interefon gamma
Cer: Ceramide
SMase: Sphingomyelinase
nSMase: neutral sphingomyelinase
SMS: Sphingomyelin synthase
ARA: Arachidonic acid
